# Evolution of copper coupons resistance in *Enterobacteriaceae* strains isolated from high-contact surfaces in hospital

**DOI:** 10.12669/pjms.40.6.8435

**Published:** 2024-07

**Authors:** Lamia Benhalima, Sandra Amri, Mourad Bensouilah

**Affiliations:** 1 Lamia Benhalima, Associate Professor. Department of Biology, University 8 Mai 1945, Guelma, Algeria; 2 Sandra Amri, Associate Professor. Department of Biology, University 8 Mai 1945, Guelma, Algeria; 3 Mourad Bensouilah, Professor Department of Marine Biology, University Badji-Mokhtar Annaba, Algeria

**Keywords:** Copper coupon, Enterobacterial strain, Resistance, Antibiotic development

## Abstract

**Objective:**

Metallic copper alloys have gained attention recently as a cutting-edge antibacterial weapon for areas where surface hygiene is crucial. The present study aimed to assess copper coupons (99% Cu) for their potential to decrease the viability of various *Enterobacteriaceae* strains from inanimate hospital surfaces.

**Methods:**

This in vitro-experimental study was conducted at the Microbiology Laboratory, Faculty of Natural and Life Sciences and Earth and Universe Sciences, University of Guelma, and Khodja Ahmed Public Hospital Establishment, Algeria, for a period of six months from January to May 2022. A total of 85 samples were collected from patient room door handles and bed rails at the government hospital in Guelma State, from which 12 enterobacterial isolates were obtained. These isolates were evaluated for susceptibility to copper and polyvinyl chloride (PVC) coupons using plate counts to determine bacterial viability after 72 hours of incubation at 37°C or room temperature (25°C). Antibiotic sensitivity testing was then carried out using a modified Kirby-Bauer disc diffusion method. Copper coupons’ ability to either select for or create antibiotic resistance is also determined.

**Results:**

Copper showed a bactericidal effect after three hours for *Serratia odorifera* and six hours for *Escherichia coli*. Whereas it was shown that within three days of selection, 83.33% of *Enterobacteriaceae* strains are capable of rapidly acquiring Cu resistance. Indeed, the increase in temperature reduced the effects of Cu (p<0.05; Student’s t-test). Antimicrobial susceptibility testing revealed that the copper-resistant bacteria were less sensitive than their predecessors. *Citrobacter freundii* strains showed the highest incidence of multidrug resistance. The most significant findings included widespread resistance to beta-lactams (100%-75%) and chloramphenicol (66.67%).

**Conclusion:**

These results suggest that prolonged copper usage may contribute to the development of antibiotic resistance, which could have significant ramifications.

## INTRODUCTION

The presence of microorganism cells on hospital surfaces may pose a major hazard for patients’ health and play a critical role in the frequency of hospital-acquired infections.^1^ Susceptible patients can contract multi-resistant pathogens from inanimate surfaces by simply touching their hands, as these adherent cells have been shown to survive for months on such surfaces.^2^ Strains of *Enterobacteriaceae*, a group of Gram-negative bacteria found in the intestinal tract of humans and animals, are frequently present in the hospital environment and capable of adhering to a variety of abiotic surfaces. Certain genera of this family can be the cause of serious nosocomial opportunistic infections.^3^

To reduce microbial contamination on surfaces frequently touched by hands, several studies have shown that the application of antimicrobial materials, mainly those containing copper, has proved effective.^4^ Copper, an essential nutrient for all living organisms, is considered the metal of choice for hospital surfaces to eliminate in a few hours several pathogenic organisms, including *Enterobacteriaceae* strains, which is described by the term contact killing.^5^ The use of copper surfaces as antimicrobial and antiadhesive materials in healthcare and biomedical devices and in high-touch surfaces in hospital equipment (such as beds and door handles) is actually a very important strategy to control and inactivate microorganisms.^1^ It was found that the copper coupons can reduce the rate of hospital-acquired infections and the rate of surface colonization with several bacteria. Copper coupons can mitigate the bacterial burden on high-touch surfaces by their ability to release copper ions, which affect the integrity of the cell membrane, inhibit essential enzymes, and degrade deoxyribonucleic acid (DNA).^2^

Given that bacteria have the capacity for horizontal gene transfer and can rapidly adapt to different stresses and become resistant to antibacterial surfaces, the persistence of antibacterial activity of copper metal surfaces remains a matter of debate. Despite promising results in controlling the bacterial load in the hospital environment through the use of copper materials, certain limitations need to be taken into account when new copper application strategies are developed, such as microbial resistance to copper and cross-resistance to antimicrobial agents. This study aims to examine the ability of *Enterobacteriaceae* isolated from hospital inanimate surfaces to survive on copper coupons 99% over time, and at different temperatures to understand whether a significant reduction in microbial burden and if there is a link to multiple antibiotic resistance.

## METHODS

This in vitro-experimental study was conducted at the Microbiology Laboratory, Faculty of Natural and Life Sciences and Earth and Universe Sciences, University of Guelma, and Khodja Ahmed Public Hospital Establishment, Guelma, Algeria, from January to May 2022. Eighty five (n = 85) inanimate surface samples were randomly collected aseptically from different patient rooms at the government hospital in Guelma State using sterile swabs, specifically from patient room door handles and bed rails. Samples were labeled and transported to the microbiology laboratory immediately. All the samples were transferred to test tubes containing sterile Tryptone Soy Broth (TSB) (Oxoid, UK), streaked onto their respective isolation media: MacConkey agar, Hektoen agar, *Salmonella-Shigella* agar, and blood agar (Oxoid, UK), and incubated aerobically for 24 hours at 37°C.^6^ The isolates of enterobacteria were identified by biochemical characteristics according to Bergey’s Manual of Systematic Bacteriology.^7^ Twelve isolated strains (n=12) belonging to the *Enterobacteriaceae* family were used throughout this study. Prior to the experiment, the cultures were activated by two successive transfers in 10 ml TSB (Oxoid, UK) for 24 h at 30°C, resulting in a culture with approximately 3.25 × 10^9^ CFU/ml.

### Inclusion criteria:

All *Enterobacteriaceae* isolates were used in the present study which included:

- *Citrobacter freundii*,
- *Klebsiella oxytoca*,
- *Proteus mirabilis*,
- *Serratia odorifera*,
- *Escherichia coli*.

### Exclusion criteria:

- Other Gram-negative strains such us *Vibrio* and *Chryseobacterium* were excluded from this study.

### Ethical Approval

The study has been approved by the Scientific Council of the Department of Ecology and Environmental Engineering, Faculty of Natural and Life Sciences and Earth and Universe Sciences, University of Guelma, on 21^st^ September 2021 (CS/DEGE/FSNVTU/UG2022), and the bioethical approval was taken from the Institutional Bioethical Committee of the Public Hospital Establishment, Khodja Ahmed, No. PHE_KA/2023/07, dated 4^th^ July 2023, Guelma, Algeria.

Copper coupons (one cm × one cm, 99% Cu) were initially soaked in acetone to remove any manufacturing process debris and grease. After cleaning, the coupons were immersed in ethanol and flamed in a Bunsen burner before being transferred to a sterile plastic container with a lid to prevent contamination before inoculation. Similar sized cleaned PVC coupons were individually placed in glass test tubes and autoclaved at 121°C for 15 minutes as controls. Coupons were aseptically inoculated with 20 µl of each bacteria culture. Following inoculation, the coupons were incubated at either room temperature (~ 25°C) or 37°C for varying periods, ranging from one to 72 hours. Control coupons were removed immediately after inoculation at time zero to determine the initial number of viable bacterial cells. After incubation, cells were removed from the coupons by vortexing for 30 s in 10 ml sterile Phosphate-Buffered Saline (PBS) (Oxoid, UK). The detached cultivable cells were enumerated on Tryptone Soy Agar (TSA) (Oxoid, UK). A volume of 100 µl was removed and serially diluted in sterile PBS to 10^-4^. TSA plates were then inoculated with 50 µl of each dilution, which was spread evenly over the surface of the agar with a sterile, glass spreader. The detection limit of this procedure was 1 Log_10_ CFU. The bacteriostatic activity was defined as a ≥2 to 3 Log_10_ CFU reduction and bactericidal activity as a ≥3 Log_10_ CFU from the initial inoculums. Two replicates were completed for each coupon sample, as well as for each time and temperature regime.^8^

The susceptibility of bacterial isolates to different antibacterial agents was determined before and after exposure to copper on Mueller-Hinton (MH) agar plates (Oxoid, UK) by the modified Kirby-Bauer disc diffusion technique.^9^ A total of nine antibiotics were used, which are as follows (Lab. Pvt. Mumbai, India): Ampicillin (AMP, 10 μg); Amoxicillin (AMX, 25U); Cefazolin (CZ, 30 μg); Ceftazidime (CAZ, 30 μg); Cefotaxime (CTX, 5 μg); Chloramphenicol (C, 30 μg); Ofloxacin (OFX, 5 μg); Tetracyclin (TET, 30 μg) and Trimethoprim (TMP, 5 μg). The reference strain *Escherichia coli* ATCC® 25922 was included in each assay for quality control.

### Statistical analysis

It was done using Statistical Package for Social Studies (SPSS; IBM, version 25.0 Corporate headquarters one New Orchard Road Armonk, New York 10504-1722 United States). The viable counts of bacteria exposed to PVC and Cu were compared using the unpaired Student’s t-test. To evaluate statistically significant differences in phenotypic characteristics within the same species, Fisher’s exact test was used. Differences were considered statistically significant when p < 0.05.

## RESULTS

A total of 85 samples were collected from hospital surfaces, with 12 samples testing positive for *Enterobacteriaceae* isolates (14.12%). Door handles were the most contaminated surfaces (58.33% of isolates), followed by bed rails (over 41% of isolates). *Enterobacteriaceae* were the most commonly detected opportunistic pathogens, with five species identified, and followed by *Vibrionaceae* (*Vibrio vulnificus*) and *Chryseobacteriaceae* (*Chryseobacterium indologens*). The predominant species recovered was *Citrobacter freundii*, accounting for 33.33% of total enterobacterial strains. No significant differences were observed in prevalence values for each species’ tested characteristics (p>0.5; Fisher’s exact test).

**Fig.1:**
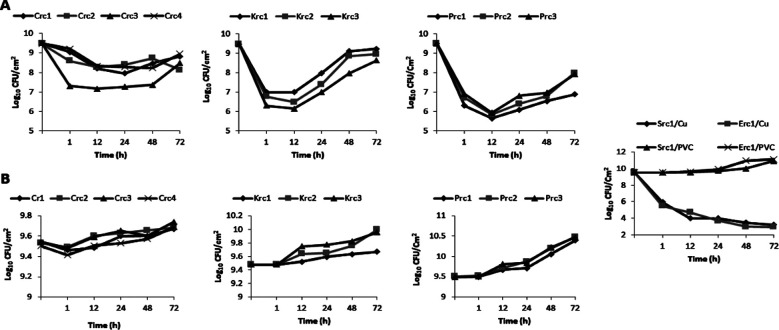
Evaluation of the growth capacity of *Enterobacteriaceae* strains on copper (A) and PVC (B) coupons at 25°C. **Crc:** *Citrobacter freundii*; Krc: *Klebsiella oxytoca*; **Prc:** *Proteus mirabilis*; **Src:** *Serratia odorifera*; **Erc:** *Escherichia coli*.

**Fig.2:**
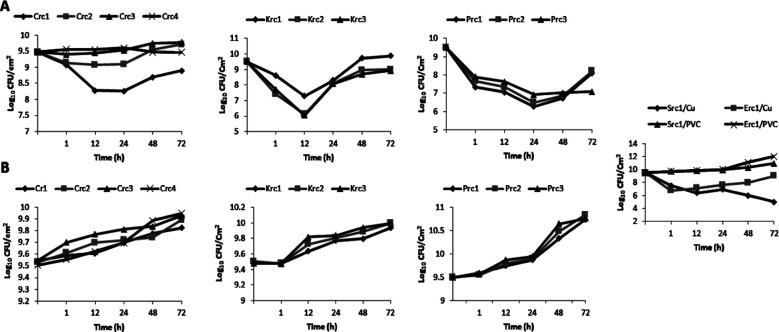
Evaluation of the growth capacity of *Enterobacteriaceae* strains on copper (A) and PVC (B) coupons at 37°C. **Crc:** *Citrobacter freundii*; **Krc:** *Klebsiella oxytoca*; Prc: *Proteus mirabilis*; **Src:** *Serratia odorifera*; **Erc:** *Escherichia coli*.

The viability of *Enterobacteriaceae* isolates was assessed after incubating them for different time periods on PVC and Cu coupons, revealing that the composition of the surfaces significantly impacts the viability of these opportunistic pathogens. Cu coupons exhibited significant antibacterial activity, resulting in a rapid decrease in viable cells of *Citrobacter freundii* to 7.97 ± 0.5 Log_10_ UFC/cm^2^ within 24 hours at 25°C and to 9.1 ± 0.4 Log_10_ UFC/cm^2^ within 12 hours at 37°C (Fig.1 and 2). The best time to decrease the viable cells of *Klebsiella oxytoca* strains was after 12 hour with a mean Log_10_ UFC/cm^2^ of approximately 6.54 ±0.4 and 6.5 ±0.6 at 25°C and 37°C, respectively. Similarly, the concentration of *Proteus mirabilis* recovered from copper coupons over time was found to be significantly different than the control surfaces (*p*-value <0.001; Student’s t test). For all *Proteus* strains, reductions in bacterial counts decreased from 9.5 ± 0.01 to 5.8 ± 0.12 Log_10_ UFC/cm^2^ on copper coupons within 12 hours at 25°C (Fig.1).

Copper coupons were more effective against *Serratia odorifera* and *E.coli* over prolonged exposure times, with significant killing observed after 72 hours at 25°C (Fig.1). However, under the same conditions, cells applied on PVC did not experience a significant loss in viability (p<0.001; Student’s t-test).

Unexpectedly, the majority of enterobacterial strains survived after 48 and 72 hours of exposure to copper coupons (Fig.1 and 2). This important time period on Cu coupons led to an increase in the survival of *Crc, Krc, and Prc* isolates. Fig.2 demonstrates that *Crc1 and Crc2* strains were able to grow at a higher load than the initial inoculums after 72 hours at 37°C. However, the value of viable *Krc2*, *Prc1*, *Prc2*, and *Prc3* cells exceeded the initial inoculums with values above 10 Log_10_ UFC/cm^2^ (Fig. 1 and 2). At room temperature (25°C), the highest mean counts for enterobacterial strains on copper coupons within 72 hours were 8.6 ± 0.3 Log_10_ UFC/cm^2^ for *Citrobacter* strains, 8.83 ± 0.3 Log_10_ UFC/cm^2^ for *Klebsiella* strains, and 7.6 ± 0.5 Log_10_ UFC/cm^2^ for *Proteus* strains (Fig. 1 and 2). It could be convincingly demonstrated that the cells clearly survived longer with high loads on the copper coupons at 37°C. A significant difference in the killing rates of bacteria grown under different temperature conditions was observed (p<0.05; Student’s t-test).

To determine the antibacterial property of copper coupons on enterobacterial strains, Δ Log_10_ CFU/cm^2^ between the starting inoculum and the viable cell count of the strains isolated at each time interval was calculated (Table-I). A bactericidal effect of Cu was shown for *Serratia odorifera* and *E. coli* isolates at the first hour of exposure. Cu reduced the initial inoculum of *Proteus mirabilis* isolates by a mean value of >2 Log_10_ CFU/cm^2^ at one hour, but a bactericidal effect was shown within 12 hours at 25°C. Against the group of *Citrobacter freundii* isolates, no bactericidal or bacteriostatic effects of Cu were recovered.

**Table-I:** Difference between the viable cell count of starting inoculums and the viable cell count of isolated strains at each time interval (Δ Log_10_ CFU/cm^2^).

Time (hours)					
***Coupon material/T°C***	**1**	**12**	**24**	**48**	**72**
**Δ Log_10_ CFU/cm^2^* for Citrobacter freundii* isolates** ^NS^					
Cu99% / 25°C	0.96	1.5	1.52	1.3	0.89
Cu99% / 37°C	0.18	0.39	0.36	0.11	0.02
PVC/ 25°C	0.07	-0.02	-0.07	-0.08	-0.17
PVC/ 37°C	-0.08	-0.14	-0.2	-0.28	-0.37
p-values*	0.000455	0.00055	0.000461	0.00036	0.000251
**Δ Log_10_ CFU/cm^2^* for Klebsiella oxytoca* isolates** ^NS^					
Cu99% / 25°C	2.78	2.94	2.02	0.83	0.54
Cu99% / 37°C	1.59	3.02	1.35	0.38	0.56
PVC/ 25°C	0.0004	-0.16	-0.19	-0.26	-0.4
PVC/ 37°C	0.014	-0.24	-0.32	-0.38	-0.49
p-values*	0.00063	0.000248	0.00026	0.000243	0.00036
**Δ Log_10_ CFU/cm^2^* for Proteus mirabilis* isolates** ^NS^					
Cu99% / 25°C	2.87	3.69	0.89	1.65	1.91
Cu99% / 37°C	2	2.17	2.95	2.64	1.71
PVC/ 25°C	-0.01	-0.24	-0.31	-0.66	-0.95
PVC/ 37°C	-0.08	-0.31	-0.41	-0.99	-1.28
p-values*	0.00031	0.00054	0.000433	0.00053	0.00061
**Δ Log_10_ CFU/cm^2^* for Serratia odorifera* isolate**					
Cu99% / 25°C	3.55	3.1	5.51	6.03	6.3
Cu99% / 37°C	1.97	3.1	2.56	3.5	4.5
PVC/ 25°C	-0.02	-0.05	-0.19	-0.49	-1.39
PVC/ 37°C	1.96	-0.32	-0.45	-0.79	-1.45
p-values*	0.00054	0.000414	0.00049	0.000732	0.000662
**Δ Log_10_ CFU/cm^2^* for Escherichia coli* isolate**					
Cu99% / 25°C	4.03	4.8	5.8	6.51	6.5
Cu99% / 37°C	2.76	2.38	1.88	1.51	0.51
PVC/ 25°C	-0.02	-0.12	-0.42	-1.44	-1.61
PVC/ 37°C	-0.22	-0.35	-0.51	-1.61	-2.52
p-values*	0.000361	0.00026	0.000264	0.00054	0.00023

Asterisks denote p-values obtained after applying Student’s t-test to compare the number of viable bacteria exposed to PVC and Cu; differences that were statistically significant in the within-species comparisons. ^NS^ denotes the absence of statistically significant differences in prevalence values associated with phenotypic characteristics for each species (*Citrobacter freundii* isolates, p= 0.252; *Klebsiella oxytoca* isolates, p= 0.278; *Proteus mirabilis*, p= 0.341; Fisher’s exact test).

All hospital isolates before exposure to copper coupons were sensitive to tetracycline and ofloxacin (Table-II). Whereas, 91.67% of isolates were susceptible to trimethoprim, and 75% of isolates were sensitive to chloramphenicol. Overall, the frequency of the antibiotics to which bacterial isolates are resistant increases after contact with copper coupons. Considering the multiple drug resistance, 83.33% of isolates became MDR (resistance to antibiotics belonging to three or more classes).

**Table-II:** Antibiotic sensitivity profiles for enterobacterial isolates.

Antibiotic	Before exposure to copper coupons				After exposure to copper coupons			

	Susceptibility		Resistance		Susceptibility		Resistance	

	n	%	n	%	n	%	n	%
AMP	1	8.33	11	91.67	0	0	12	100
AMX	1	8.33	11	91.67	0	0	12	100
CZ	2	16.66	10	83.33	1	8.33	11	91.67
CAZ	4	33.33	8	66.67	2	16.66	10	66.67
CTX	7	58.33	5	41.67	2	16.66	10	66.67
C	9	75	3	25	4	33.33	8	66.67
OFX	12	100	0	0	6	50	6	50
TET	12	100	0	0	6	50	6	50
TMP	11	91.67	1	8.33	7	58.33	5	41.67

## DISCUSSION

The dissemination of healthcare-associated infections is complex and has multifactorial causes, leading to notable patient morbidity and mortality. The data reported in this study established that the hospital’s high-contact surfaces were highlighted as a potential reservoir of enterobacterial strains. *Citrobacter freundii*, *Klebsiella oxytoca*, and *Proteus mirabilis* were the most isolated species and this is in accordance with other studies.^10^,^11^

The current study demonstrates a significant effect of copper coupons on *E. coli* and *Serratia odorifera* over time. Nevertheless, copper also displays potent biocidal activity against *Citrobacter freundii*, *Klebsiella oxytoca*, and *Proteus mirabilis* after initial exposure periods (1-24 hours). This result may be explained by the fact that copper has an initial temporary toxic or bacteriostatic effect on bacterial strains during an initial period by hindering their multiplication.^12^ On its turn, Souli et al. demonstrated copper coupons antibacterial activities on multidrug-resistant Gram-negative pathogens especially with copper coupons containing 99% Cu, which had a bactericidal effect within six hour for *E.coli*.^13^ In addition, Champagne et al. showed that bacterial inactivation by copper surfaces was more complete after two hour of exposure.^14^

Unfortunately, our results show that the impact of copper on enterobacterial strains disappears after longer incubation periods. The microbial burden recovered from the sampled copper coupons was well above the postulated environmental risk threshold for hospital-associated infection acquisition (2.7 Log_10_ CFU). This is in line with a previous study where the antimicrobial effects of copper were limited and the cells of some of the Gram-negative bacterial strains survived on copper surfaces for 48 hours or more.^15^ As reported by Benhalima et al., the clinical isolates *Citrobacter freundii and Klebsiella oxytoca* exhibited resistance to heavy metals such as copper.^16^ In Gram-negative bacteria, several control systems of copper homeostasis have been reported: generation of a less toxic form, export of copper to the extracellular environment with copper-ATPase export pumps, and sequestration of copper in the cytoplasm.^17^

Temperature is a major environmental parameter to be taken into account in multi-stress studies. In the present study, at both temperatures (25 °C and 37 °C), the antibacterial properties of copper coupons on *Enterobacteriaceae* strains are affected (*p*-value <0.05; Student’s t-test). The increase in temperature after Cu exposure promoted the development of all strains. This might suggest that thermal stress-tolerant species might additionally be more Cu-tolerant. These results are consistent with those of Lambert, who has shown that temperature could modulate the structural and functional effects of Cu on microbial communities.^18^ Additionally, he has suggested that a rise in temperature could affect how sensitive microbial populations are to copper, regardless of whether they have had past exposure to the metal on a long-term basis or not.

The emergence of antimicrobial resistance among *Enterobacteriaceae* species and the role of the environment in hosting and transmitting these organisms have become a global public health concern.^19^,^20^ It is noteworthy here that after exposure to copper coupons, the majority of isolates have become multi-drug resistant (MDR). This suggests that there has been a co-selection of metallic copper resistance and resistance against antibiotics. This observation is supported by Pietsch et al. who reported that horizontal gene transfer of copper resistance as well as other genes were associated with antibiotic resistance in *Enterobacteriaceae* strains isolated from different sources.^21^ The reason behind the resistance capacity of enterobacterial strains may be efflux pump systems that help to survive on copper coupons; these efflux pump systems were involved in co-selection via a cross-resistance mechanism, which consequently develops bacterial antibiotic resistance systems.^22^ Therefore, a deeper understanding of this relationship is necessary to stop the emergence of antibiotic resistance and ultimately treatment failures.

The current study is more clinically significant since it demonstrates that antimicrobial copper surfaces are not equally effective in lowering the ambient microbial load isolated from the hospital environment. This is most likely owing to bacterial strains’ propensity to acquire antibiotic resistance, which is both startling and concerning.

### Limitations

This study involved a number of bacteria isolated from inanimate surfaces in a single hospital. Well-designed studies involving a large bacterial population and different surfaces in several hospitals are needed to effectively assess the resistance of bacterial strains to copper coupons, even after prolonged contact. However, a big step has to be taken to transform the experimental studies into a real clinical environment. Further studies are needed to evaluate the role of copper resistance as a selective force in antibiotic resistance maintenance.

## CONCLUSION

According to previous results, resistance to copper coupons was observed after three days in *Klebsiella oxytoca* and *Proteus mirabilis* strains. The increase in temperature may have selected more tolerant species. Furthermore, the selection of copper-antibiotic cross-resistance in all isolated enterobacteria indicates a major concern about the continuing and future usage of copper antimicrobial surfaces.

### Authors’ Contribution:

**LB:** Conception of the idea, design, statistical analysis, interpretation, editing of the manuscript, responsible for the accuracy and integrity of the study.

**LB, SA:** Data collection and manuscript writing.

**LB, MB:** Conceptualization, methodology and writing revisions.

**LB, SA & MB:** Review and final approval of manuscript.
